# Metaviromic Characterization of Betaflexivirus Populations Associated with a *Vitis* cultivar Collection in South Africa

**DOI:** 10.3390/v15071474

**Published:** 2023-06-29

**Authors:** David A. Read, Genevieve D. Thompson, Dirk Z. H. Swanevelder, Gerhard Pietersen

**Affiliations:** 1 Department of Biochemistry, Genetics and Microbiology, Forestry and Agricultural Biotechnology Institute (FABI), University of Pretoria, Pretoria 0002, South Africa; 2 Gene Vantage, 53 Kyalami Boulevard, Kyalami Business Park, Johannesburg 1684, South Africa; genevieve@gene-vantage.com; 3 Agricultural Research Council (ARC)—Biotechnology Platform, 100 Old Soutpan Road, Onderstepoort, Pretoria 0110, South Africa; swanevelderd@arc.agric.za; 4 Patho Solutions, Olifantskop Road, Wellington 7655, South Africa; gerhard@pathsol.co.za

**Keywords:** South Africa, viticulture, grapevine cultivars, grapevine viruses, betaflexiviruses

## Abstract

South Africa is associated with a centuries-old viticultural industry, accompanied by a diverse range of wine and table grape cultivars and an extensive history of pervasive introductions of vine material and associated viruses. The *Vitis* D2 collection in Stellenbosch represents the most comprehensive collection of *Vitis* species, hybrids, and cultivars in South Africa. We collected leaf petiole material from 229 accessions from this collection. Our metaviromic analyses revealed a total of 406 complete/near complete genomes of various betaflexiviruses. Among these, we identified the presence of grapevine rupestris stem pitting-associated virus and grapevine viruses A, B, E, F, H (GVH), I (GVI), and M (GVM). Notably, this study marks the first report of GVH, GVI, and GVM in South Africa, which were confirmed via RT-PCR. This research significantly contributes to our understanding of viral diversity and introductions in South African viticulture and emphasizes the need for vigilant monitoring and management of viral infections. Our findings lay the groundwork for strategies that mitigate the impact of viruses on South Africa’s wine industry, which generates an annual revenue of approximately 500 million USD.

## 1. Introduction

*Vitis vinifera* L. (grapevine) is one of the most important perennial crops and has an extensive history of domestication and global trade. Grapevines are vegetatively propagated through vine cuttings that promote virus accumulation, which may cause stunted growth, reduced yield, poor fruit quality, and even vine death. Recent advancements in high-throughput sequencing (HTS) technologies have revealed unprecedented levels of viral diversity, with close to 90 known viruses infecting grapevine [1]. Understanding virus diversity is crucial for developing effective management strategies to control spread and minimize their impact on crop yield and quality, as well as identifying new viruses that may emerge and threaten grapevine production in the future.

Members of the family *Betaflexiviridae* are common in grapevine and include vitiviruses (Genus: *Vitivirus*), foveaviruses (Genus: *Foveavirus*), trichoviruses (Genus: *Trichovirus*), and carlaviruses (Genus: *Carlavirus*). Vitiviruses are positive-sense (+ssRNA) viruses with ~7500 nucleotides (nt) genomes that typically express five gene products, including replication-associated proteins (RAPs), the “22-kDa” transmission-associated protein, movement protein, coat protein (CP), and a nucleic acid-binding protein that functions as a suppressor of RNA silencing [2]. *Vitivirus* is also one of the most diverse of the grapevine-infecting genera with thirteen accepted/proposed species, that includes *Grapevine virus A* (GVA) [3], *Grapevine virus B* (GVB) [4], *Grapevine virus D* (GVD) [5], *Grapevine virus E* (GVE) [6], *Grapevine virus F* (GVF) [7], *Grapevine virus G* (GVG) [8], *Grapevine virus H* (GVH) [9], *Grapevine virus I* [10], *Grapevine virus J* [11], *Grapevine virus K* [12], grapevine virus L (GVL) [13], grapevine virus M (GVM) [14], grapevine virus N (GVN), and grapevine virus O (GVO) [15]. 

Grapevine vitiviruses are common in viral populations of South African vineyards [16]. GVA, GVB, and GVE are widespread, with GVA and GVB showing diverse populations [17,18,19], while GVE has more homogenous populations [20]. GVF, GVL, and two novel vitiviruses, i.e. GVN and GVO, were recently identified at the same vineyard as this study [15,21,22]. When vitiviruses are present as single infections they are generally associated with very mild symptoms [23]. However, synergistic co-infections with other viruses, in particular leafroll-associated viruses, result in a number of economically important vineyard diseases [24]. Multiple infections with grapevine rupestris stem pitting-associated virus (GRSPaV, genus: *Foveavirus*) is relatively common since GRSPaV is one of the most widespread and diverse grapevine-infecting viruses [25]. GRSPaV particles (~720 nm) encapsidate +ssRNA genomes of ~8700 nt in length that encode five gene products, including the replicase complex, three movement-associated proteins of the triple-gene block (TGB), and the CP [26]. GVA, GVB, and GRSPaV are part of a complex of viruses associated with rugose wood (RW) [7,27]—a grapevine disease of global economic importance [28]. In South Africa, GVA is associated with Shiraz disease [18] and GVB with corky bark disease [19]. Except for GRSPaV, GVA, GVB, and GVD, the contributions of grapevine betaflexiviruses to disease expression still requires confirmation with Koch’s postulates [29]. The fulfilment of Koch’s postulates is complicated in that betaflexiviruses are rarely observed as single isolates in populations [23], with the fulfillment of these criteria necessitating the production of infectious clones [30]. Here, a comprehensive South African collection of *Vitis* species, hybrids, and cultivars are analyzed to provide new insights into the diversity of grapevine betaflexiviruses and represents the first report of GVH, GVI, and GVM in the country.

## 2. Materials and Methods

### 2.1. Collection of Plant Material

In December 2019, petioles from a total of 229 samples were collected from the vineyard D2 *Vitis* cultivar collection at the Agricultural Research Council’s Nietvoorbij Campus, Stellenbosch, South Africa. Cultivars were planted in replicates within the vineyard. Petiole samples were collected from these cultivar replicates (1 to 5) (Table S1), combined, and subsequently treated as single samples for each cultivar/accession. Samples were stored at 4 °C for several days until extraction. 

### 2.2. Isolation of Total RNA and RNAtag-Seq Library Preparation

Petioles were removed and 0.5 g of each sample was macerated in extraction bags (Bioreba, Reinach, Switzerland) prior to total RNA isolation using the cetyltrimethylammonium bromide protocol of White et al. [31]. RNA quality and quantity were determined with a NanoPhotometer N60 (Implen, Munich, Germany) and a Qubit 3 (RNA Broad Range assay, Thermo Fisher Scientific, Waltham, MA, USA). Purified RNA (300 ng) from each sample was used to generate RNAtag-seq libraries in accordance with Shishkin et al. [32]. Up to 32 samples were tagged and then pooled into single libraries. The 229 samples were sequenced via eight such pooled libraries. Sequencing was performed using an Illumina HiSeq 2500 instrument (Illumina, San Diego, CA, USA; ARC—Biotechnology Platform, Onderstepoort, Pretoria, South Africa), as paired-ends (2 × 125 nt) on separate lanes, using TruSeq V4 chemistry (Illumina, San Diego, CA, USA). 

### 2.3. Bioinformatics of RNAtag-Seq Data

Initial quality control of raw sequence reads was performed using FastQC (Babraham Bioinformatics, Cambridge, UK) prior to demultiplexing into individual sample datasets with Je software [33]. Trimming was performed using CLC Genomics Workbench 9 (Qiagen Bioinformatics, Aarhus, Denmark) with parameters described previously [21]. De novo assembly was carried out using metaSPAdes 3.14.0 [34]. The identities of viral contigs were determined using both blastn and blastx [35] against the viral subset of the NCBI nucleotide and protein databases. Contigs showing homology to betaflexiviruses were selected for further analyses and deposited into GenBank. The location of open reading frames (ORF) was identified using ORF finder [36]. Contigs containing a complete RNA-dependent RNA polymerase (RdRP) ORF or at least 80% in length of the complete sequence available on the GenBank were considered to be complete/partially complete. 

### 2.4. Phylogenetic Analyses of Vitivirus Replicase Associated Protein (RAP) Sequences

The derived amino acid sequences of the RAP of each virus were aligned against the cognate sequences of other extant variants of the same species obtained from GenBank, using BioEdit 7.2.5 [37]. GRSPaV references were selected according to Tobar et al. [25] and each cluster named according to the proposed nomenclature of Meng and Rowhani [38]. Best-fit maximum likelihood (ML) models were determined using MEGA X [39]. Branch support was estimated using 1000 bootstrap replicates with the same model parameters for all phylogenies. The following best-fit ML models were used, with the respective viruses indicated in brackets: Jones–Taylor–Thornton (JTT) [40] with empirical base frequencies (F), invariant sites (I) and gamma distribution G (n = 5) (GVA), JTT + G + I (GRSPaV, GVB, GVH), Le Gascuel (LG) [41] + G (GVE, GVF, GVI, GVM).

### 2.5. RT-PCR Confirmation of Selected Vitiviruses

RT-PCR targeting the coat protein coding region (Table 1) was used to confirm the presence of GVH, GVI, and GVM in five randomly selected samples for each virus. Two-step RT-PCR reactions were carried out using Promega GoScript^TM^ Reverse Transcriptase and GoTaq^®^ Taq polymerase (Promega, Madison, WI, USA), according to manufacturer’s instructions. Amplicons were visualized on 1.5% agarose gels following electrophoresis. The amplicons from 45-07 Red Globe, 04-13 Alphonse Lavallee, 05-04 Bacchus, and 24-15 Optenhorst were chosen to represent GVF, GVH, GVI, and GVM, respectively, and subjected to Sanger sequencing (Inqaba Biotechnical Industries, Pretoria, South Africa).

## 3. Results and Discussion

### 3.1. Provenance of Collected Samples

All samples were collected from a decades-old vineyard. The D2 *Vitis* cultivar collection took place at ARC’s Nietvoorbij Campus, Stellenbosch. Samples comprised 212 *V. vinifera* cultivars, including primarily wine grape, but also some table grape cultivars. Additionally, seventeen *V. labrusca* cultivars, non-vinifera species, and interspecific hybrids were also included (Table S1). Originally, cultivars were planted as five individual vine replicates, but over time a reduced number of these survived, resulting in one to five replicates per cultivar. All vines showed symptoms of virus-like disease at the time of collection, including decline, leaf rolling, and leaf reddening or yellowing. These symptoms are consistent grapevine leafroll-associated virus 3 (GLRaV3) infections that occur ubiquitously in vineyards in South Africa [42]. 

### 3.2. Illumina Sequencing and Data Analysis

**Figure 1:**
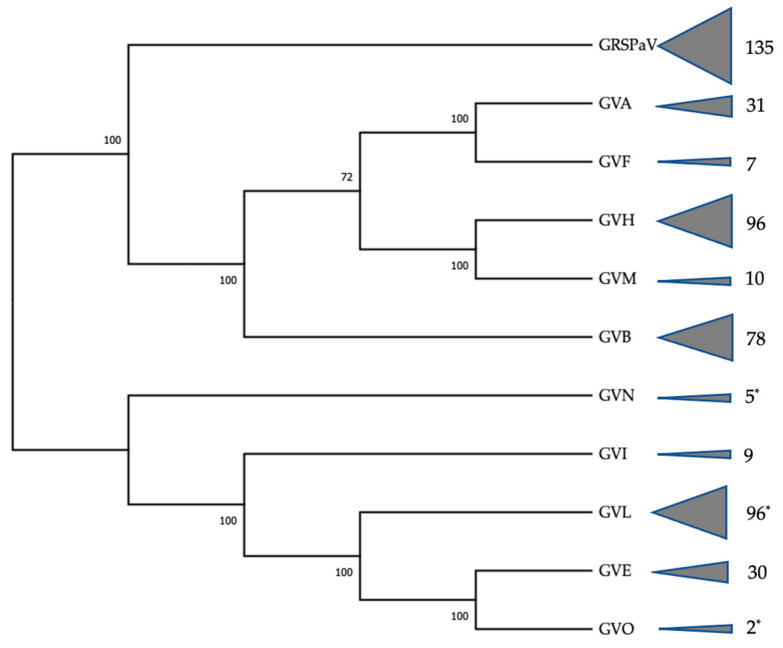
Betaflexivirus diversity is associated with the samples from this study. Endpoint triangles are scaled to the number of complete/near complete genomes associated with each virus branch, with the actual virus number next to it. * Data for GVL, GVN, and GVO are presented by Read et al. [14,21].

Pre- and post-trim read numbers were 934,919,526 and 872,021,325, respectively, with an average of 3,807,953 post-trimmed reads per dataset and a range of 918,017 to 11,363,736 cross samples (Table S1). Datasets are available in NCBI’s sequence read archive (SRA, PRJNA626577). The Biosample accession number of each individual dataset is presented in Table S1. Blastn analysis of the resulting metaSPAdes-derived contigs indicated the presence of several different grapevine leafroll-associated viruses, grapevine virus L (GVL) [22], grapevine virus N (GVN), and O (GVO) [15], as well as six different viroids [43]. Additional betaflexiviruses were identified, namely GRSPaV, GVA, GVB, GVE, GVF, GVH, GVI, and GVM. A phylogenetic representation of the betaflexivirus diversity, together with the numbers of each variant associated with the D2 vineyard, is shown in Figure 1.

### 3.3. Viruses Identified: GRSPaV

GRSPaV is a member of the genus *Foveavirus* and was the only foveavirus detected during this study. A total of 135 complete/near complete GRSPaV genomes were assembled, which were derived from 100 of the 229 accessions analyzed—a cultivar positivity rate of at least 45% (Table S1). The complete genomes had an average length of 8639 nt and a modal length of 8727 nt. The high levels of diversity associated with GRSPaV in other countries [44] was highlighted by the phylogenetic analysis that indicated the presence of all seven known subgroups with the following numbers of each: I—37, IIa—19, IIb—22, IIc—5, IId—14, III—32, IV—1. (Figure S1). The majority of cultivars evaluated were associated with a single variant of GRSPaV, however 26 populations had more than one and up to four different variants. Glasa et al. [45] demonstrated a similar propensity for GRSPaV to accumulate as multiple variants within individual grapevines. However, the pooling of grapevine replicates into a single sample for processing may be a contributing factor to the observed patterns in this study. Complete genome nucleotide sequence identity between variants ranged between 66–100% in this study (Figure S2). 

GRSPaV has previously been reported in South Africa [20,46] and can be classified into at least seven subgroups [45,46]. It is one of the most frequently reported viruses in commercial vineyards and has pronounced genetic diversity [44]. Our findings support these prior studies [47]. It is also consistently reported with the greatest prevalence at the regional level [48], with an incidence of over 50% in some surveys [49,50]. GRSPaV is one of several viruses that form the RW complex, which results in distortions of the woody cylinder of the grapevine, leading to symptoms of pitting and grooving on the scion and/or rootstock [26] and is one of the most important disease phenotypes of grapevine [28]. RW can be further classified into several syndromes, which include: Rupestris stem pitting (RSP), Kober stem grooving (KSG), LN 33 stem grooving (LNSG), and grapevine corky bark (GCB) [27].

### 3.4. GVA

In this study, 31 of the 229 (13.5%) samples were associated with GVA and produced complete genomes. This is similar to the positivity rate described among commercial vineyards in South Africa [51]. Genome sizes ranged from 6296 to 7382 nt, with an average and modal length of 7212 and 7361 nt, respectively. GVA variants from South Africa cluster into four phylogroups, with variants from phylogroup II being associated with Shiraz disease symptoms [18]. The RdRP amino acid phylogeny generated here appears to resolve all four phylogroups known from South Africa (Figure S3). Only one variant clustered within phylogroup II, while 19, 16, and variants clustered within I, III, and IV, respectively. This indicates that for the case of the cultivar collection at least, the Shiraz disease (SD)-inducing variant was exceedingly rare. Variants’ nucleotide sequence identity for these samples ranged between 59–99% (Figure S4).

As with most of the viruses discussed in this study, GVA is a member of the *Vitivirus genus* [52]. Like many vitiviruses, single infections result in mild-to-no symptoms [23], however synergistic co-infections, particularly with grapevine leafroll-associated viruses, often lead to damaging disease symptoms [24]. GVA is part of the RW complex [7] and in South Africa results in a particular syndrome known as Shiraz disease [18]. GVA is considered to be a particularly diverse vitivirus that groups into four distinct phylogroups, with a highly heterogeneous population even at the regional level [53]. GVA diversity has been studied in South Africa, albeit at the single gene level [18] and the distribution of the virus is well understood [51]. However, complete genome data for GVA in South Africa and global data are lacking. This study confirms GVA heterogeneity in South Africa and also shows high levels of diversity even at the single vineyard level, possibly due to the diverse global origins of the collection material.

### 3.5. GVB

GVB is part of the RW complex and is most commonly associated with GCB symptoms [19] and graft incompatibility. Here, 93 complete/near complete GVBs were generated from 78/229 (34%) of the cultivars evaluated. The ML phylogeny showed that variants from this study were part of a diverse population of GVBs and clustered within phylogroups with all extant variants with complete genome data, except GVB 248 from South Africa and GVB-BIB-BR and ISA-BR from Brazil (Figure S5). The majority of variants, however, clustered with GVB H-1 and 94/971 from South Africa and GVB-QMWH from China, which are considered to be highly divergent [54]. Genome sizes ranged from 7307 to 7615 nt, with an average and modal length of 7563 and 7601 nt, respectively. Average nucleotide identity (ANI) between variants from this study ranged between 72–99% (Figure S6). The high diversity associated with GVA and GVB variants from this study, as illustrated by the phylogenies in Figures S3 and S5, is consistent with other findings, where no geographic clustering has been observed [28]. This is most likely the result of long-term, intensive global trade of vegetatively propagated grapevine-planting material. 

### 3.6. GVE

The phylogeny indicates that GVE sequences from this study cluster with isolate SA94 from South Africa [20], with the exception of the genome derived from 02–01 *V. flexuisa,* which clustered with sequences from Croatia, Japan, Greece, and China (Figure S7). This was confirmed by average nucleotide identity figures, which indicated shared identities of 92–99% among GVE variants from this study, with the exception of 02–01 *V. flexuisa* GVE, which shared 64–69% ANI with other variants (Figure S8). Genome sizes ranged from 6181 to 7573 nt, with an average and modal length of 7453 and 7562 nt, respectively. Although GVE was detected in 13% of the accessions considered, with 30 genomes, the high level of homology relative to GVA and GVB may indicate a more recent introduction of the virus into South Africa. The roles that GRSPaV, GVA, and GVB play in the manifestation of RW-associated symptoms are well established; while the disease etiology of more recently identified vitiviruses, such as GVE, GVG, GVH, GVI, GVL, GVN, and GVO, are not yet known, it is likely that they contribute to the manifestation of RW-like symptoms in grapevines [28], although GVG is unlikely to contribute to these symptoms in grapevines [55]. GVE was first described from grapevines in Japan [6], and soon after the complete genome of a variant from South Africa was characterized [20]. A total of 29 complete/near complete genomes were generated for GVE. 

### 3.7. GVF

GVF incidences in the study were low, with only seven genomes being recovered. In addition to the initial detection of GVF in South Africa [51], the complete genome of a divergent variant was also determined (V5; KP114220) [21]. Three of the variants from this study grouped with V5, while the remaining four grouped to form a unique phylogroup (Figure S9). ANI values for GVF variants from this study varied between 76–99% (Figure S10). GVF was originally characterized from a grapevine accession in California [7]. Since then, it has been detected in geographically disparate regions, including South Africa [51], Tunisia [56], Iran [57], Greece [58], Pakistan [59], Russia [60], and Croatia [61]. While it has been suggested that GVF contributes to RW associated disease phenotypes [29], no definitive disease etiology has been determined for GVF. 

### 3.8. GVH

GVH was the betaflexivirus with the second highest incidence from this study at 42% (96/229), only behind GRSPaV at 45%. The phylogenetic analysis of the RdRP of the variants from this study showed that the majority group within a single phylogroup together with other variants from Portugal and USA, except for the variant from 44-05 which formed a unique phylogroup (Figure S11). This indicates a highly homogenous GVH population from samples in this study. Pairwise homology analysis confirmed this, showing that variants from this study share 94–99% nucleotide identity (Figure S12). The minimum genome length was 7214 nt and maximum length was 7496 nt. The average and modal lengths were 7440 and 7446 nt. This is the first time that GVH is being reported from South Africa. 

GVH is a recently described vitivirus, initially discovered in Portugal [9]. Since then it has been found in other Mediterranean countries including Greece [62], Croatia [63], and Italy [64], as well as California and Tennessee in the United States [65,66] and Russia [60]. In most previous surveys, GVH has been detected at low levels of incidence. Jagunic et al. [63] showed that GVH was present in 8% of cultivars from sampled from Croatia. Schianchi et al. [64] detected GVH in a single plant of 38 plants tested from Italy, with Shvets et al. [60] detecting GVH in 3 of 43 cultivars from Russia. Conversely, Sabaghian et al. [29] reported that GVH was the most prevalent virus (29.5%) among grapevine samples in Iran.

### 3.9. GVI

GVI is a recently discovered vitivirus (originally described from New Zealand) and is most closely related to GVE [10]. The only other countries from which GVI has been reported is Greece and USA [67], making South Africa the fourth country to report its presence. The Greek variant appears to be divergent when compared with that of the New Zealand variant. A total of nine (4%) complete genomes were derived from cultivars in this study. The phylogeny suggests that these variants are closely related. They also cluster within a single phylogroup, together with the Greek variant (Figure S13). This supports the hypothesis that GVI groups into two separate phylogenetic groups [67]. ANI values for GVI variants were between 98 and 99% (Figure S14). Interestingly, two of the Greek cultivars analyzed in this study, namely Agostenga and Assyrtiko, were associated with GVI. These also grouped with the Greek variant D2-1/19 (Figure S13), possibly indicating an introduction of GVI through one or both of these cultivars. The average genome length is 7496 nt, similar to the complete genome sequence of 7507 nt, as described by Blouin et al. [10]. 

### 3.10. GVM

A putatively novel vitivirus, called GVM, was recently detected and described by Alabi et al. [14] from the Blanc du Bois hybrid variety in Texas and was shown to be closely related to GVH. Eleven complete genomes were generated for GVM from as many different cultivars. Ten of these were associated with *Vitis vinifera* cultivars and one with *V. champinii.* GVM is reported here for the first time, in South Africa and on *V. vinifera*. The phylogeny shows that the ten *V. vinifera* GVM variants group within a phylogroup, distinct from that formed by the Texan variant TX-WAT (Figure S15). The variant from *V. champinii* also forms an isolated phylogroup, suggesting that it is highly divergent. The complete genome ANI shared between this variant and TX-WAT was 70% (Figure S16). The average amino acid identity was 78% for the RdRP and 87% for the CP genes, which is just below the cusp of the currently accepted species demarcation thresholds for the *Vitivirus* genus [68]. While the *V. champinii* variant of GVM may represent a putatively novel vitivirus species, its detection from only a single sample suggests that additional variants need to be identified before this can be confirmed, especially given that the sequence is on the edge of the demarcation threshold. 

### 3.11. Evidence for Mixed Infections

Multiple betaflexivirus infections were identified in 139 cultivars from this study, based on the recovery of complete/near complete genomes. Up to six different betaflexiviruses were identified within single accessions, and 50 unique combinations were observed (Table 2), with GRSPaV-GVL being the most common with ten occurrences. The specific combinations of betaflexivirus populations for each cultivar are presented in Table S2. It is important to note that the evidence for mixed infections in this study is likely to be inflated due to the initial pooling of multiple cultivar replicates per sample.

## 4. Conclusions

This study is one of the most comprehensive investigations into the diversity of betaflexiviruses in grapevines and provides significant advancements in our understanding of this family’s diversity within the South African context. It also marks the first formal reports of GVH, GVI, and GVM in the country. While the exact disease etiologies of these viruses are still unclear, their potential roles in the expression of RW symptoms should not be disregarded when considering grapevine phytosanitation. 

Similar to many grapevine germplasm collections worldwide, the D2 vineyard at Nietvoorbij clearly consists of materials collected over a long period from various global sources, and as such includes a diverse range of viruses. Previous studies by Read et al. [15,22] and Morgan et al. [43] have contributed to our understanding of the virology of this vineyard. The findings of this study confirm previous observations, including the high diversity and incidence of GRSPaV and the presence of the IId phylogroup, as initially identified by Mostert et al. [46]. Additionally, this study also confirms a high diversity of GVA and GVB without any geographical clustering, as well as homogeneity of GVE in South Africa, as initially shown by Coetzee et al. [20]. Multiple virus infections were found to be common, with numerous unique combinations identified (Table 2).

Ampelographic collections hold significant value as they often embody a history of extensive cultivar introductions. These collections can serve as sentinels for the early detection of phytopathogens and provide materials for the development of detection techniques. In addition to this, grapevine biodiversity is currently facing significant threats and undergoing a widespread contraction, partly due to the “globalization of wine” phenomenon [69]. This trend has resulted in the global cultivation of a limited number of popular grape cultivars, such as Cabernet Sauvignon, Merlot, and Chardonnay [70]. Ampelographic collections like the D2 vineyard play a crucial role in preserving grapevine biodiversity and serve as valuable genetic resources [60]. However, the presence and accumulation of viruses in this collection and others in Russia [58] and Croatia [71] for example continues to diminish their value. In order to effectively combat viruses, it is crucial to have a comprehensive understanding of their presence, which requires continuous monitoring, as exemplified by the practices conducted at the vine collection of University of California Davis [72]. While techniques like thermotherapy and meristem tip culture have shown promise in eliminating GVA [73], there is limited information available regarding the elimination of recently described vitiviruses. Future research should address this gap.

Metaviromics studies, like the one conducted here, will remain instrumental in successfully implementing grapevine phytosanitary measures in grape-growing regions. By expanding our knowledge of the viral landscape, these studies contribute to the development of effective virus elimination strategies, which are crucial for preserving grapevine health and biodiversity.

## Figures and Tables

**Table 1:** RT-PCR coat protein gene primers for confirmation of GVH, GVI, and GVM. The sequence, genomic target region, annealing temperature (Ta), and product size (RT-PCR) are listed. Key: GVF—grapevine virus F; GVH—grapevine virus H; GVI—grapevine virus I; GVM—grapevine virus M; CP—coat protein; nt—nucleotide.

Virus	Primer Name	Sequence (5′–3′)	Target (nt)	Ta °C	Product (bp)
GVF	GVF-CP-F	CTACTCTTGTTATGCCAGAGGTCTA	6450–6474	52	507
	GVF-CP-R	AATCAAACATGACCTGCGGTTCT	6935–6957		
GVH	GVH-CP-F	GCTATGATGTGCCTATGTATCTCGAA	6564–6589	53	424
	GVH-CP-R	ACGAGAATTCAGACCTTGGATCACA	6964–6988		
GVI	GVI-CP-F	GGAGATAAGGAAGGCAGTCCTACA	6420–6443	55	485
	GVI-CP-R	GCCTCAGATCGAGTGAGTTTACC	6883–6905		
GVM	GVM-CP-F	TTACATTGCTGTGGTGGGCACTTCGA	6571–6596	58	391
	GVM-CP-R	AGAGTCGTGAATTTAGCCCCTGGATC	6937–6962		

**Table 2:** Unique combinations of betaflexivirus infections and number of occurrences.

Unique Betaflexivirus Combination	Number of Occurrences
GRSPaV, GVA, GVF, GVH, GVI, GVL	1
GVA, GVB, GVE, GVH, GVM, GVL	2
GVA, GVB, GVH, GVM, GVL	1
GRSPaV, GVB, GVH, GVI, GVN	1
GRSPaV, GVB, GVH, GVI, GVL	1
GRSPaV, GVB, GVE, GVH, GVL	1
GRSPaV, GVA, GVE, GVH	1
GRSPaV, GVA, GVH, GVI	1
GRSPaV, GVE, GVH, GVL	1
GRSPaV, GVB, GVI, GVL	1
GRSPaV, GVB, GVE, GVH	1
GRSPaV, GVB, GVH, GVN	1
GRSPaV, GVA, GVH, GVL	2
GRSPaV, GVA, GVE, GVH	1
GVA, GVE, GVF, GVL	1
GVA, GVB, GVH, GVL	2
GRSPaV, GVB, GVF, GVH	1
GRSPaV, GVB, GVH, GVL	3
GRSPaV, GVB, GVE, GVL	2
GVA, GVB, GVE, GVL	2
GRSPaV, GVB, GVH, GVL	3
GRSPaV, GVA, GVH, GVL	2
GRSPaV, GVA, GVB, GVL	1
GRSPaV, GVB, GVH, GVL	4
GRSPaV, GVB, GVF, GVH	1
GRSPaV, GVB, GVL, GVN	1
GVB, GVH, GVL	7
GRSPaV, GVI, GVL	1
GRSPaV, GVH, GVI	1
GRSPaV, GVB, GVH	5
GVA, GVB, GVI	1
GVA, GVB, GVH	2
GRSPaV, GVH, GVL	8
GRSPaV, GVE, GVH	3
GVH, GVL, GVN	1
GVE, GVH, GVL	4
GVA, GVB, GVF	1
GVA, GVB, GVE	1
GRSPaV, GVB, GVM	2
GRSPaV, GVH, GVL	8
GRSPaV, GVB, GVH	4
GVA, GVH, GVL	1
GRSPaV, GVL	10
GVA, GVL	1
GVB, GVH	9
GVB, GVE	3
GRSPaV, GVB	6
GVH, GVL	9
GVE, GVL	4
GVH, GVI	1

## Data Availability

All RNAseq datasets are available at National Center for Biotechnology Information’s (NCBI) Sequence Read Archive (SRA), accession PRJNA626577. All assembled sequences have been submitted to NCBI GenBank, listed in Table S1.

## Supplementary Materials

The following supporting information can be downloaded at: https://www.mdpi.com/article/10.3390/v15071474/s1, Figure S1: RNA-dependent RNA polymerase (RdRP) gene phylogeny of GRSPaV variants; Figure S2: pairwise ANI values shared between GRSPaV variants; Figure S3: RNA-dependent RNA polymerase (RdRP) gene phylogeny of GVA variants; Figure S4: pairwise ANI values shared between GVA variants; Figure S5: RNA-dependent RNA polymerase (RdRP) gene phylogeny of GVB variants; Figure S6: pairwise ANI values shared between GVB variants; Figure S7: RNA-dependent RNA polymerase (RdRP) gene phylogeny of GVE variants; Figure S8: pairwise ANI values shared between GVE variants; Figure S9: RNA-dependent RNA polymerase (RdRP) gene phylogeny of GVF variants; Figure S10: pairwise ANI values shared between GVF variants; Figure S11: RNA-dependent RNA polymerase (RdRP) gene phylogeny of GVH variants; Figure S12: pairwise ANI values shared between GVH variants; Figure S13: RNA-dependent RNA polymerase (RdRP) gene phylogeny of GVI variants; Figure S14: pairwise ANI values shared between GVI variants; Figure S15: RNA-dependent RNA polymerase (RdRP) gene phylogeny of GVM variants; Figure S16: pairwise ANI values shared between GVM variants; Table S1: sample information, number of reads, and BioSample and GenBank accession numbers for each accession; Table S2: mixed infections for cultivar accessions with more than one betaflexivirus present.
