# Supplementary material for: Metaviromic Characterization of Betaflexivirus Populations Associated with a Vitis cultivar Collection in South Africa
Source: Viruses. 2023 Jun 29;15(7):1474. doi: 10.3390/v15071474 (PMC10385141; doi:10.3390/v15071474)
Supplement: Supplementary file 1 [file viruses-15-01474-s001.zip › Figure S1.pdf]

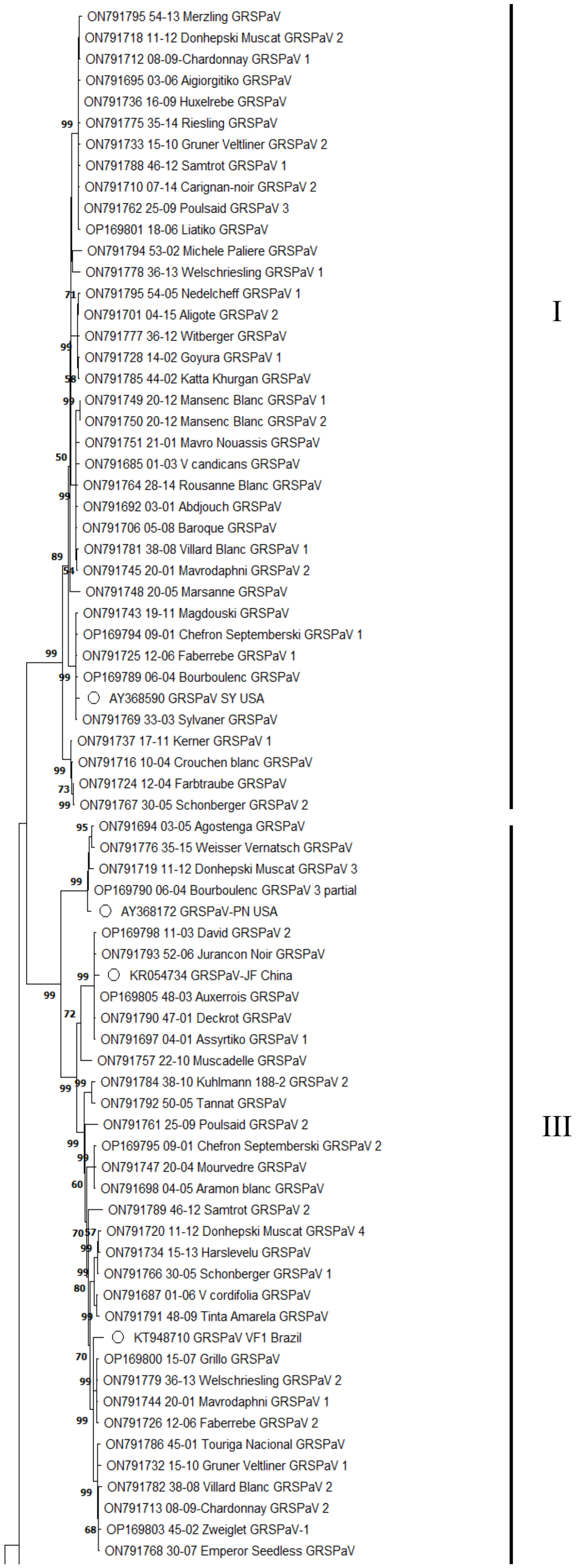

ON791795 54-13 Merzling GRSPaV
ON791718 11-12 Donhepski Muscat GRSPaV 2
ON791712 08-09-Chardonnay GRSPaV 1
ON791695 03-06 Aigiorgitiko GRSPaV
ON791736 16-09 Huxelrebe GRSPaV
ON791775 35-14 Riesling GRSPaV
ON791733 15-10 Gruner Veltliner GRSPaV 2
ON791788 46-12 Samtrot GRSPaV 1
ON791710 07-14 Carignan-noir GRSPaV 2
ON791762 25-09 Poulsaid GRSPaV 3
OP169801 18-06 Liatiko GRSPaV
ON791794 53-02 Michele Paliere GRSPaV
ON791778 36-13 Welschriesling GRSPaV 1
ON791795 54-05 Nedelcheff GRSPaV 1
ON791701 04-15 Aligote GRSPaV 2
ON791777 36-12 Witberger GRSPaV
ON791728 14-02 Goyura GRSPaV 1
ON791785 44-02 Katta Khurgan GRSPaV
ON791749 20-12 Mansenc Blanc GRSPaV 1
ON791750 20-12 Mansenc Blanc GRSPaV 2
ON791751 21-01 Mavro Nouassis GRSPaV
ON791685 01-03 V candicans GRSPaV
ON791764 28-14 Rousanne Blanc GRSPaV
ON791692 03-01 Abdjouch GRSPaV
ON791706 05-08 Baroque GRSPaV
ON791781 38-08 Villard Blanc GRSPaV 1
ON791745 20-01 Mavrodaphni GRSPaV 2
ON791748 20-05 Marsanne GRSPaV
ON791743 19-11 Magdouski GRSPaV
OP169794 09-01 Chefron Septemberski GRSPaV 1
ON791725 12-06 Faberrebe GRSPaV 1
OP169789 06-04 Bourboulenc GRSPaV
AY368590 GRSPaV SY USA
ON791769 33-03 Sylvaner GRSPaV
ON791737 17-11 Kerner GRSPaV 1
ON791716 10-04 Crouchen blanc GRSPaV
ON791724 12-04 Farbtraube GRSPaV
ON791767 30-05 Schonberger GRSPaV 2
ON791694 03-05 Agostenga GRSPaV
ON791776 35-15 Weisser Vernatsch GRSPaV
ON791719 11-12 Donhepski Muscat GRSPaV 3
OP169790 06-04 Bourboulenc GRSPaV 3 partial
AY368172 GRSPaV-PN USA
OP169798 11-03 David GRSPaV 2
ON791793 52-06 Jurancon Noir GRSPaV
KR054734 GRSPaV-JF China
OP169805 48-03 Auxerrois GRSPaV
ON791790 47-01 Deckrot GRSPaV
ON791697 04-01 Assyrtiko GRSPaV 1
ON791757 22-10 Muscadelle GRSPaV
ON791784 38-10 Kuhlmann 188-2 GRSPaV 2
ON791792 50-05 Tannat GRSPaV
ON791761 25-09 Poulsaid GRSPaV 2
OP169795 09-01 Chefron Septemberski GRSPaV 2
ON791747 20-04 Mourvedre GRSPaV
ON791698 04-05 Aramon blanc GRSPaV
ON791789 46-12 Samtrot GRSPaV 2
ON791720 11-12 Donhepski Muscat GRSPaV 4
ON791734 15-13 Harslevelu GRSPaV
ON791766 30-05 Schonberger GRSPaV 1
ON791687 01-06 V cordifolia GRSPaV
ON791791 48-09 Tinta Amarela GRSPaV
KT948710 GRSPaV VF1 Brazil
OP169800 15-07 Grillo GRSPaV
ON791779 36-13 Welschriesling GRSPaV 2
ON791744 20-01 Mavrodaphni GRSPaV 1
ON791726 12-06 Faberrebe GRSPaV 2
ON791786 45-01 Touriga Nacional GRSPaV
ON791732 15-10 Gruner Veltliner GRSPaV 1
ON791782 38-08 Villard Blanc GRSPaV 2
ON791713 08-09-Chardonnay GRSPaV 2
OP169803 45-02 Zweiglet GRSPaV-1
ON791768 30-07 Emperor Seedless GRSPaV
I
III

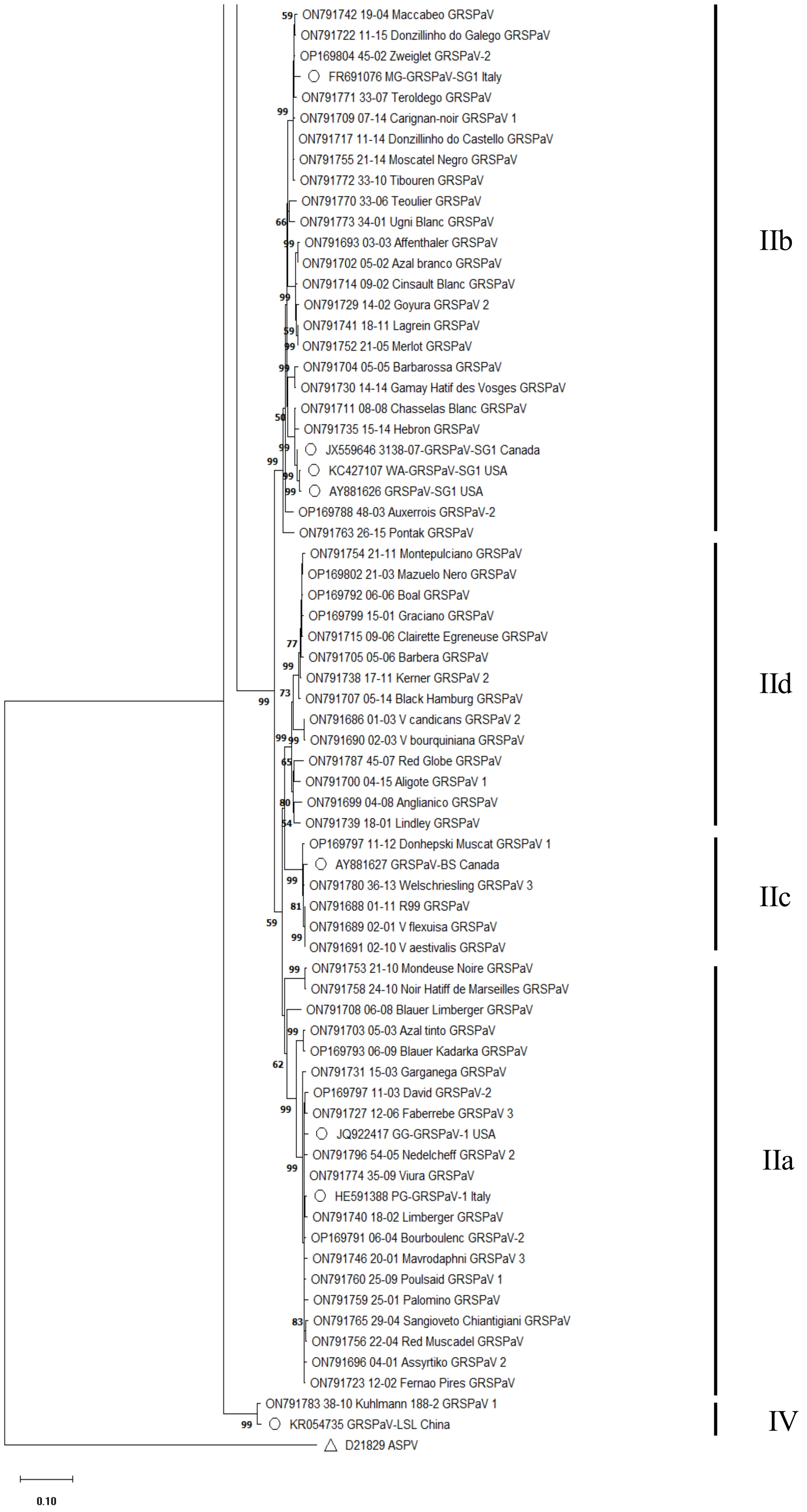

Figure S1: Maximum likelihood phylogeny based on the amino acid sequences of the RNA-dependant RNA polymerase (RdRp) gene of grapevine rupestris stem pitting-associated virus (GRSPaV) from this study together the cognate sequences from selected variants of GRSPaV from GenBank (indicated by open circles). The cognate RdRp sequence from apple stem pitting virus was used as an outgroup (indicated by an open triangle). The phylogeny represents the tree with the highest log likelihood and was generated in MEGA X (Stecher et al. 2020) using the Jones-Taylor-Thornton (JTT) (Jones, W. R. Taylor, and J. M. Thornton. (1992) with gamma distribution (n=5) and invariant sites. Bootstrapping was applied (1000 replicates) and the percentage of trees in which the associated taxa clustered together is shown next to the branches. Bootstrap percentages lower than 50 are not shown.
