# Supplementary material for: Metaviromic Characterization of Betaflexivirus Populations Associated with a Vitis cultivar Collection in South Africa
Source: Viruses. 2023 Jun 29;15(7):1474. doi: 10.3390/v15071474 (PMC10385141; doi:10.3390/v15071474)
Supplement: Supplementary file 1 [file viruses-15-01474-s001.zip › Figure S10.pdf]

		1	2	3	4	5	6	7
06-08_Blauer_Limberger_GVF	1		6601	6966	6074	6094	6094	6089
33-05_Tempranillo_GVF	2	87,55		7063	5726	5733	5735	5739
45-07_Red_Globe_GVF	3	92,39	93,76		6057	6060	6068	6070
11-05_Delight_GVF	4	80,56	75,94	80,33		6896	6882	6886
14-14_Gamay_Hatif_des_Vosges_GVF	5	80,82	76,03	80,37	91,47		7434	7457
22-10_Muscadelle_GVF	6	80,82	76,06	80,48	91,29	98,61		7487
24-10_Noir_Hatiff_de_Marseilles_GVF	7	80,76	76,18	80,58	91,34	98,91	99,31	

Figure S10: Average nucleotide identity comparisons between the complete/near complete genomes of grapevine virus F variants from this study. The x-axis values express average nucleotide variability between variants as a percentage, while the y-axis values present the total number of nucleotide differences between each variant.
