# Supplementary material for: Metaviromic Characterization of Betaflexivirus Populations Associated with a Vitis cultivar Collection in South Africa
Source: Viruses. 2023 Jun 29;15(7):1474. doi: 10.3390/v15071474 (PMC10385141; doi:10.3390/v15071474)
Supplement: Supplementary file 1 [file viruses-15-01474-s001.zip › Figure S11.pdf]

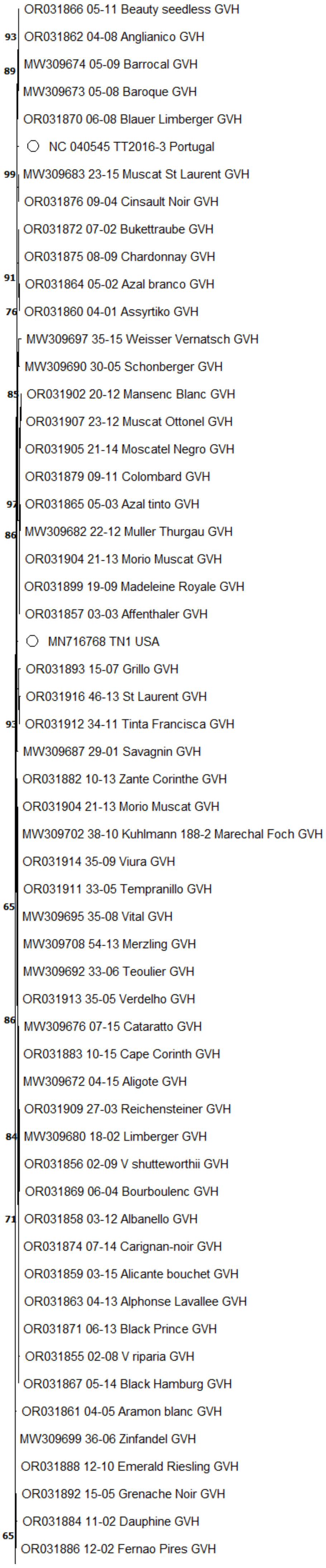
OR031866 05-11 Beauty seedless GVH
93
OR031862 04-08 Anglianico GVH
89
MW309674 05-09 Barrocal GVH
MW309673 05-08 Baroque GVH
OR031870 06-08 Blauer Limberger GVH
NC 040545 TT2016-3 Portugal
99
MW309683 23-15 Muscat St Laurent GVH
OR031876 09-04 Cinsault Noir GVH
OR031872 07-02 Bukettraube GVH
OR031875 08-09 Chardonnay GVH
91
OR031864 05-02 Azal branco GVH
76
OR031860 04-01 Assyrtiko GVH
MW309697 35-15 Weisser Vernatsch GVH
MW309690 30-05 Schonberger GVH
85
OR031902 20-12 Mansenc Blanc GVH
OR031907 23-12 Muscat Ottonel GVH
OR031905 21-14 Moscatel Negro GVH
OR031879 09-11 Colombard GVH
97
OR031865 05-03 Azal tinto GVH
86
MW309682 22-12 Muller Thurgau GVH
OR031904 21-13 Morio Muscat GVH
OR031899 19-09 Madeleine Royale GVH
OR031857 03-03 Affenthaler GVH
MN716768 TN1 USA
OR031893 15-07 Grillo GVH
OR031916 46-13 St Laurent GVH
93
OR031912 34-11 Tinta Francisca GVH
MW309687 29-01 Savagnin GVH
OR031882 10-13 Zante Corinthe GVH
OR031904 21-13 Morio Muscat GVH
MW309702 38-10 Kuhlmann 188-2 Marechal Foch GVH
OR031914 35-09 Viura GVH
OR031911 33-05 Tempranillo GVH
65
MW309695 35-08 Vital GVH
MW309708 54-13 Merzling GVH
MW309692 33-06 Teoulier GVH
OR031913 35-05 Verdelho GVH
86
MW309676 07-15 Cataratto GVH
OR031883 10-15 Cape Corinth GVH
MW309672 04-15 Aligote GVH
OR031909 27-03 Reichensteiner GVH
84
MW309680 18-02 Limberger GVH
OR031856 02-09 V shutteworthii GVH
OR031869 06-04 Bourboulenc GVH
71
OR031858 03-12 Albanello GVH
OR031874 07-14 Carignan-noir GVH
OR031859 03-15 Alicante bouchet GVH
OR031863 04-13 Alphonse Lavallee GVH
OR031871 06-13 Black Prince GVH
OR031855 02-08 V riparia GVH
OR031867 05-14 Black Hamburg GVH
OR031861 04-05 Aramon blanc GVH
MW309699 36-06 Zinfandel GVH
OR031888 12-10 Emerald Riesling GVH
OR031892 15-05 Grenache Noir GVH
OR031884 11-02 Dauphine GVH
65
OR031886 12-02 Fernao Pires GVH

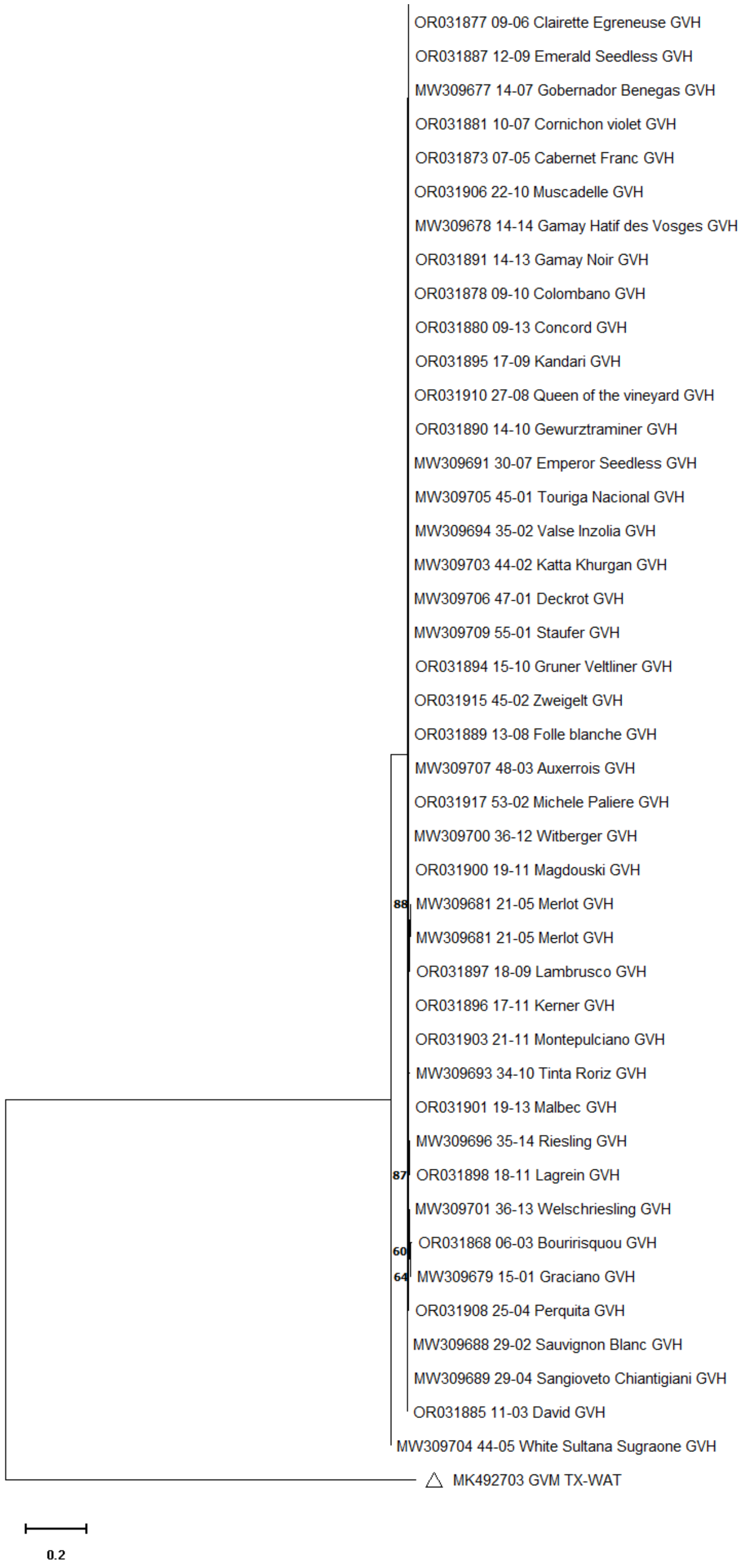

Figure S11: Maximum likelihood phylogeny based on the amino acid sequences of the RNA-dependant RNA polymerase (RdRp) gene of grapevine virus H (GVH) from this study together the cognate sequences from selected variants of GVH from GenBank (indicated by open circles). The cognate RdRp sequence from grapevine virus M was used as an outgroup (indicated by an open triangle). The phylogeny represents the tree with the highest log likelihood and was generated in MEGA X (Stecher et al. 2020) using the Jones-Taylor-Thornton (JTT) (Jones, W. R. Taylor, and J. M. Thornton. (1992) with gamma distribution (n=5) and invariant sites. Bootstrapping was applied (1000 replicates) and the percentage of trees in which the associated taxa clustered together is shown next to the branches. Bootstrap percentages lower than 50 are not shown.
