# Supplementary material for: Metaviromic Characterization of Betaflexivirus Populations Associated with a Vitis cultivar Collection in South Africa
Source: Viruses. 2023 Jun 29;15(7):1474. doi: 10.3390/v15071474 (PMC10385141; doi:10.3390/v15071474)
Supplement: Supplementary file 1 [file viruses-15-01474-s001.zip › Figure S14.pdf]

		1	2	3	4	5	6	7	8	9
07-05_Cabernet_Franc_GVI	1		7407	7413	7408	7409	7411	7345	7413	7412
05-08_Baroque_GVI	2	98,69		7413	7416	7419	7414	7346	7411	7418
05-06_Barbera_GVI	3	98,77	98,77		7446	7445	7447	7379	7445	7450
05-03_Azal_tinto_GVI	4	98,71	98,81	99,21		7471	7447	7381	7445	7455
04-01_Assyrtiko_GVI	5	98,72	98,85	99,20	99,56		7448	7385	7449	7456
05-04_Bacchus_GVI	6	98,75	98,79	99,23	99,24	99,25		7416	7456	7460
04-05_Aramon_blanc_GVI	7	97,87	97,88	98,32	98,36	98,45	98,83		7398	7394
06-08_Blauer_Limberger_GVI	8	98,77	98,75	99,20	99,21	99,31	99,36	98,69		7468
03-05_Agostenga_GVI	9	98,76	98,84	99,27	99,33	99,35	99,40	98,52	99,51	

Figure S14: Average nucleotide identity comparisons between the complete/near complete genomes of grapevine virus I variants from this study. The x-axis values express average nucleotide variability between variants as a percentage, while the y-axis values present the total number of nucleotide differences between each variant.
