# Supplementary material for: Metaviromic Characterization of Betaflexivirus Populations Associated with a Vitis cultivar Collection in South Africa
Source: Viruses. 2023 Jun 29;15(7):1474. doi: 10.3390/v15071474 (PMC10385141; doi:10.3390/v15071474)
Supplement: Supplementary file 1 [file viruses-15-01474-s001.zip › Figure S16.pdf]

		1	2	3	4	5	6	7	8	9	10	11
01-04_V_champinii_GVM	1		5234	5223	5228	5222	5222	5223	5219	5222	5223	5225
21-06_Merseguera_GVM	2	70,16		7375	7385	7391	7294	7307	7275	7279	7283	7291
07-15_Cataratto_GVM	3	70,01	98,86		7407	7413	7296	7307	7278	7279	7283	7291
03-15_Alicante_bouchet_GVM	4	70,08	98,99	99,30		7423	7302	7317	7286	7290	7294	7301
11-14_Donzillinho_do_Castello_GVM	5	70,00	99,08	99,38	99,52		7303	7320	7286	7290	7294	7300
36-06_Zinfandel_GVM	6	70,00	97,77	97,81	97,90	97,91		7393	7358	7362	7366	7374
28-14_Rousanne_Blanc_GVM	7	70,01	97,95	97,96	98,10	98,14	99,12		7416	7418	7422	7428
24-15_Optenhorst_GVM	8	69,96	97,52	97,61	97,68	97,68	98,65	99,42		7431	7435	7437
26-14_Pontak_GVM	9	70,00	97,57	97,59	97,73	97,73	98,70	99,45	99,62		7439	7443
23-15_Muscat_St_Laurent_GVM	10	70,01	97,63	97,64	97,79	97,79	98,75	99,50	99,68	99,73		7445
28-13_Rotgipfler_GVM	11	70,04	97,73	97,75	97,88	97,87	98,86	99,58	99,71	99,79	99,81	

Figure S16: Average nucleotide identity comparisons between the complete/near complete genomes of grapevine virus M variants from this study. The x-axis values express average nucleotide variability between variants as a percentage, while the y-axis values present the total number of nucleotide differences between each variant.
