# Supplementary material for: Metaviromic Characterization of Betaflexivirus Populations Associated with a Vitis cultivar Collection in South Africa
Source: Viruses. 2023 Jun 29;15(7):1474. doi: 10.3390/v15071474 (PMC10385141; doi:10.3390/v15071474)
Supplement: Supplementary file 1 [file viruses-15-01474-s001.zip › Figure S4.pdf]

		1	2	3	4	5	6	7	8	9	10	11	12	13	14	15	16	17	18	19	20	21	22	23	24	25	26	27	28	29	30	31	32	33	34	35	36
55-01_Staufer_GVA	1		5615	5644	5618	5410	5620	5425	5269	5680	5629	5630	5629	5641	5610	5606	5631	5648	5658	5661	5616	4446	5181	5206	4447	5192	5179	5186	5183	5190	5189	5190	4368	4322	5034	5136	5072
12-02_Fernao_Pires_GVA	2	76,32		7303	7304	5470	5669	5471	5290	5710	5699	5679	5704	5709	5663	5683	5701	5687	5616	5700	5724	4405	5169	5166	4404	5199	5204	5202	5213	5206	5217	5198	4368	4351	5056	5156	5081
20-12_Mansenc_Blanc_GVA	3	76,72	99,27		7307	5469	5678	5470	5290	5708	5699	5677	5679	5684	5643	5679	5702	5693	5620	5693	5719	4405	5154	5156	4404	5190	5196	5188	5198	5198	5204	5190	4368	4351	5049	5145	5077
06-08_Blauer_Limberger_GVA	4	76,36	99,28	99,32		5470	5680	5471	5297	5703	5695	5677	5679	5685	5639	5684	5702	5688	5616	5692	5717	4405	5152	5157	4404	5180	5184	5181	5192	5186	5197	5180	4368	4351	5038	5140	5074
11-05_Delight_GVA2	5	73,58	74,36	74,34	74,36		5460	5467	5318	5500	5503	5514	5543	5558	5519	5518	5528	5485	5524	5521	5541	4412	4956	4953	4382	4951	4936	4959	4950	4953	4951	4965	4357	4335	4826	4914	4920
33-06_Teoulier_GVA-2	6	76,43	77,06	77,18	77,21	74,28		5570	5367	5779	5756	5740	5805	5809	5768	5814	5749	5739	5805	5773	5784	4439	5171	5185	4445	5195	5190	5217	5217	5212	5200	5210	4399	4356	5047	5121	5094
11-05_Delight_GVA	7	73,77	74,37	74,35	74,37	76,87	75,76		5526	5703	5627	5658	5657	5668	5666	5710	5660	5670	5702	5703	5682	4403	4955	4982	4414	4999	4990	5018	5012	5011	4983	4998	4371	4307	4839	4952	4948
52-06_Jurancon_Noir	8	71,67	71,91	71,90	72,01	74,79	73,01	77,70		5739	5683	5680	5679	5683	5747	5690	5702	5711	5683	5688	5719	4365	4858	4845	4339	4845	4827	4846	4843	4852	4830	4834	4349	4257	4766	4823	4813
03-04_Agawam_GVA	9	77,25	77,61	77,59	77,52	74,80	78,59	77,55	78,05		6119	6165	6148	6157	6159	6193	6148	6211	6210	6193	6211	4411	5180	5156	4418	5152	5150	5171	5168	5172	5185	5163	4366	4294	5015	5164	5088
48-09_Tinta_Amarela_GVA-2	10	76,55	77,46	77,46	77,41	74,84	78,28	76,52	77,29	83,22		6189	6176	6187	6151	6172	6189	6223	6205	6196	6219	4459	5195	5212	4436	5218	5202	5213	5217	5218	5222	5194	4381	4301	5017	5131	5054
17-07_Kadarka_GVA	11	76,58	77,19	77,16	77,16	74,99	78,06	76,94	77,26	83,84	84,17		6159	6173	6226	6222	6196	6189	6201	6194	6243	4423	5173	5194	4410	5176	5160	5178	5173	5174	5173	5178	4381	4334	5076	5195	5125
07-02_Bukettraube-GVA	12	76,55	77,53	77,19	77,19	75,38	78,95	76,92	77,23	83,61	83,99	83,76		7292	6211	6199	6248	6254	6227	6225	6267	4446	5188	5166	4424	5200	5166	5191	5192	5189	5195	5203	4412	4347	5060	5174	5099
45-07_Red_Globe_GVA-2	13	76,72	77,60	77,26	77,27	75,59	79,00	77,07	77,29	83,73	84,14	83,95	99,17		6218	6215	6250	6260	6240	6236	6278	4445	5188	5161	4428	5203	5167	5194	5195	5190	5194	5200	4411	4348	5062	5175	5099
26-02_Petit_Sirah_Durif_GVA	14	76,31	76,97	76,70	76,65	75,24	78,45	77,24	78,36	83,76	83,65	84,68	84,47	84,56		6310	6208	6272	6213	6241	6269	4440	5184	5152	4430	5204	5202	5202	5200	5206	5217	5194	4425	4335	5030	5145	5083
07-15_Cataratto_GVA	15	76,23	77,24	77,18	77,25	75,03	79,06	77,63	77,37	84,21	83,93	84,61	84,29	84,51	85,80		6183	6284	6262	6258	6293	4429	5164	5151	4430	5195	5179	5189	5188	5192	5183	5198	4414	4348	5025	5151	5079
20-04_Mourvedre_GVA2	16	76,52	77,46	77,47	77,47	75,12	78,12	76,91	77,48	83,54	84,10	84,20	84,90	84,93	84,36	84,01		6247	6281	6254	6264	4453	5210	5207	4437	5206	5185	5205	5207	5197	5208	5203	4402	4330	5025	5172	5067
22-15_Mourisco_de_Semente_GVA	17	76,51	77,03	77,11	77,04	74,30	77,74	76,81	77,36	84,14	84,30	83,84	84,72	84,80	84,96	85,11	84,62		6273	6308	6273	4427	5184	5166	4393	5189	5163	5180	5181	5181	5206	5189	4410	4333	5069	5138	5096
23-01_Muscat_d_Alexandrie_blanc_GVA	18	76,93	76,34	76,39	76,34	75,11	78,93	77,53	77,27	84,43	84,36	84,31	84,66	84,84	84,47	85,13	85,35	84,98		6311	6294	4438	5173	5169	4431	5171	5168	5180	5174	5178	5182	5165	4424	4325	5008	5149	5071
18-02_Limberger_GVA	19	77,00	77,48	77,38	77,37	75,08	78,51	77,55	77,37	84,22	84,26	84,25	84,66	84,81	84,89	85,10	84,98	85,45	85,81		6551	4433	5196	5161	4414	5187	5177	5206	5200	5194	5180	5191	4422	4331	5037	5157	5091
10-06_Cornifesto_GVA	20	76,38	77,80	77,74	77,71	75,36	78,66	77,26	77,78	84,47	84,58	84,90	85,23	85,38	85,26	85,57	85,12	84,98	85,57	89,09		4462	5219	5141	4412	5176	5170	5192	5185	5187	5203	5194	4431	4336	5023	5142	5113
14-13_Gamay_Noir_GVA	21	60,37	59,79	59,78	59,79	61,94	60,29	61,81	61,84	59,89	60,54	60,06	60,37	60,35	60,44	60,13	60,41	59,87	60,24	60,20	60,58		6344	5307	5175	5287	5274	5294	5302	5285	5268	5252	4887	4838	4835	4957	4950
23-15_Muscat_St_Laurent_GVA	22	70,33	70,14	69,93	69,91	67,29	70,19	67,28	65,96	70,31	70,52	70,22	70,42	70,42	70,37	70,09	70,67	70,10	70,21	70,53	70,84	86,21		6182	5176	6178	6149	6180	6199	6166	6153	6134	4893	4847	5656	5773	5682
03-03_Affenthaler_GVA	23	70,68	70,09	69,96	69,97	67,26	70,39	67,64	65,79	70,00	70,76	70,51	70,13	70,07	69,94	69,92	70,63	69,86	70,15	70,07	69,79	72,10	83,97		5270	6165	6150	6174	6167	6172	6193	6148	4877	4890	5682	5794	5739
05-04_Bacchus_GVA	24	60,38	59,77	59,76	59,77	61,52	60,37	61,96	61,46	59,99	60,23	59,88	60,07	60,12	60,30	60,14	60,20	59,41	60,15	59,93	59,90	80,32	70,31	71,59		5415	5442	5454	5453	5460	5460	5432	4881	4825	4811	4924	4898
03-15_Alicante_bouchet_GVA	25	70,49	70,54	70,42	70,28	67,23	70,53	67,88	65,79	69,94	70,84	70,27	70,59	70,64	70,65	70,52	70,62	70,17	70,18	70,42	70,27	71,82	83,92	83,75	73,56		7063	7131	7133	7129	6862	6853	4892	4841	5672	5799	5711
21-10_Mondeuse_Noire_GVA	26	70,31	70,61	70,50	70,34	67,03	70,46	67,75	65,55	69,92	70,62	70,05	70,13	70,15	70,62	70,30	70,33	69,82	70,14	70,28	70,19	71,65	83,52	83,55	73,93	95,95		7203	7232	7271	6894	6863	4903	4841	5662	5784	5707
11-03_David_GVA	27	70,40	70,58	70,39	70,30	67,34	70,83	68,13	65,81	70,20	70,77	70,30	70,47	70,51	70,62	70,44	70,60	70,05	70,30	70,68	70,49	71,92	83,94	83,87	74,09	96,88	97,85		7302	7291	6871	6860	4913	4845	5680	5806	5717
21-03_Mazuelo_Nero_GVA	28	70,36	70,73	70,53	70,45	67,22	70,83	68,05	65,77	70,16	70,83	70,23	70,49	70,53	70,59	70,42	70,63	70,06	70,22	70,59	70,39	72,03	84,20	83,78	74,08	96,90	98,25	99,20		7322	6888	6871	4915	4845	5683	5809	5712
33-06_Teoulier_GVA	29	70,46	70,64	70,53	70,37	67,26	70,76	68,04	65,89	70,21	70,84	70,24	70,45	70,46	70,68	70,48	70,50	70,06	70,28	70,51	70,42	71,80	83,75	83,85	74,17	96,85	98,78	99,05	99,47		6906	6892	4918	4850	5678	5804	5718
45-07_Red_Globe_GVA	30	70,45	70,79	70,61	70,52	67,23	70,59	67,66	65,59	70,39	70,89	70,23	70,53	70,51	70,83	70,35	70,65	70,40	70,33	70,32	70,64	71,57	83,58	84,13	74,17	93,22	93,66	93,34	93,57	93,82		6911	4885	4856	5673	5790	5693
20-04_Mourvedre_GVA	31	70,46	70,53	70,42	70,28	67,42	70,73	67,86	65,64	70,09	70,51	70,30	70,64	70,59	70,51	70,56	70,58	70,17	70,10	70,47	70,51	71,35	83,32	83,52	73,79	93,10	93,23	93,19	93,34	93,63	93,89		4874	4835	5645	5782	5679
04-05_Aramon_blanc_GVA	32	59,37	59,35	59,34	59,35	61,24	59,81	61,42	61,67	59,34	59,55	59,55	59,97	59,96	60,29	59,99	59,79	59,71	60,12	60,11	60,23	75,85	66,46	66,25	76,15	66,46	66,61	66,74	66,77	66,81	66,36	66,21		5012	4968	5062	5045
10-15_Cape_Corinth_GVA	33	58,75	59,12	59,11	59,12	60,93	59,23	60,53	60,37	58,37	58,46	58,91	59,09	59,10	59,07	59,09	58,81	58,67	58,77	58,87	58,94	75,09	65,84	66,43	75,27	65,77	65,77	65,82	65,82	65,89	65,97	65,68	78,51		5114	5241	5223
09-06_Clairette_Egreneuse_GVA	34	68,42	68,68	68,58	68,43	65,60	68,59	65,77	65,32	68,16	68,18	69,00	68,77	68,80	68,37	68,28	68,24	68,62	68,04	68,47	68,27	65,68	76,82	77,18	65,35	77,04	76,91	77,15	77,19	77,13	77,06	76,68	67,59	69,58		6181	6132
48-09_Tinta_Amarela	35	69,80	70,04	69,89	69,82	66,79	69,60	67,30	65,56	70,18	69,73	70,60	70,32	70,33	69,92	70,00	70,23	69,55	69,96	70,09	69,88	67,33	78,41	78,70	66,88	78,77	78,57	78,86	78,91	78,84	78,65	78,54	68,87	71,31	84,10		6529
33-07_Teroldego_GVA	36	68,95	69,04	68,97	68,94	67,64	69,26	68,01	66,18	69,16	68,70	69,67	69,31	69,31	69,27	69,03	68,82	69,00	68,91	69,21	69,50	68,01	77,18	77,96	67,29	77,58	77,53	77,67	77,60	77,68	77,34	77,15	69,42	71,87	83,44	88,83	

Figure S4: Average nucleotide identity comparisons between the complete/near complete genomes of grapevine virus A variants from this study. The x-axis values express average nucleotide variability between variants as a percentage, while the y-axis values present the total number of nucleotide differences between each variant.
