# Supplementary material for: Metaviromic Characterization of Betaflexivirus Populations Associated with a Vitis cultivar Collection in South Africa
Source: Viruses. 2023 Jun 29;15(7):1474. doi: 10.3390/v15071474 (PMC10385141; doi:10.3390/v15071474)
Supplement: Supplementary file 1 [file viruses-15-01474-s001.zip › Figure S5.pdf]

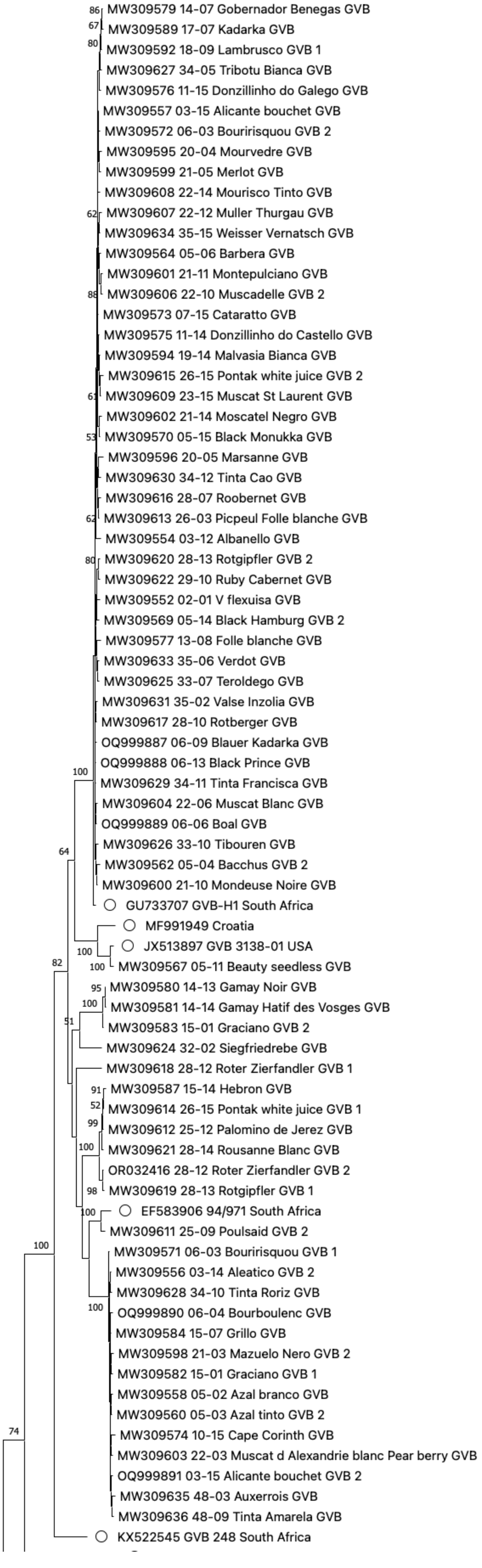
MW309579 14-07 Gobernador Benegas GVB
MW309589 17-07 Kadarka GVB
MW309592 18-09 Lambrusco GVB 1
MW309627 34-05 Tribotu Bianca GVB
MW309576 11-15 Donzillinho do Galego GVB
MW309557 03-15 Alicante bouchet GVB
MW309572 06-03 Bouririsquou GVB 2
MW309595 20-04 Mourvedre GVB
MW309599 21-05 Merlot GVB
MW309608 22-14 Mourisco Tinto GVB
MW309607 22-12 Muller Thurgau GVB
MW309634 35-15 Weisser Vernatsch GVB
MW309564 05-06 Barbera GVB
MW309601 21-11 Montepulciano GVB
MW309606 22-10 Muscadelle GVB 2
MW309573 07-15 Cataratto GVB
MW309575 11-14 Donzillinho do Castello GVB
MW309594 19-14 Malvasia Bianca GVB
MW309615 26-15 Pontak white juice GVB 2
MW309609 23-15 Muscat St Laurent GVB
MW309602 21-14 Moscatel Negro GVB
MW309570 05-15 Black Monukka GVB
MW309596 20-05 Marsanne GVB
MW309630 34-12 Tinta Cao GVB
MW309616 28-07 Roobernet GVB
MW309613 26-03 Picpeul Folle blanche GVB
MW309554 03-12 Albanello GVB
MW309620 28-13 Rotgipfler GVB 2
MW309622 29-10 Ruby Cabernet GVB
MW309552 02-01 V flexuisa GVB
MW309569 05-14 Black Hamburg GVB 2
MW309577 13-08 Folle blanche GVB
MW309633 35-06 Verdot GVB
MW309625 33-07 Teroldego GVB
MW309631 35-02 Valse Inzolia GVB
MW309617 28-10 Rotberger GVB
OQ999887 06-09 Blauer Kadarka GVB
OQ999888 06-13 Black Prince GVB
MW309629 34-11 Tinta Francisca GVB
MW309604 22-06 Muscat Blanc GVB
OQ999889 06-06 Boal GVB
MW309626 33-10 Tibouren GVB
MW309562 05-04 Bacchus GVB 2
MW309600 21-10 Mondeuse Noire GVB
GU733707 GVB-H1 South Africa
MF991949 Croatia
JX513897 GVB 3138-01 USA
MW309567 05-11 Beauty seedless GVB
MW309580 14-13 Gamay Noir GVB
MW309581 14-14 Gamay Hatif des Vosges GVB
MW309583 15-01 Graciano GVB 2
MW309624 32-02 Siegfriedrebe GVB
MW309618 28-12 Roter Zierfandler GVB 1
MW309587 15-14 Hebron GVB
MW309614 26-15 Pontak white juice GVB 1
MW309612 25-12 Palomino de Jerez GVB
MW309621 28-14 Rousanne Blanc GVB
OR032416 28-12 Roter Zierfandler GVB 2
MW309619 28-13 Rotgipfler GVB 1
EF583906 94/971 South Africa
MW309611 25-09 Poulsaid GVB 2
MW309571 06-03 Bouririsquou GVB 1
MW309556 03-14 Aleatico GVB 2
MW309628 34-10 Tinta Roriz GVB
OQ999890 06-04 Bourboulenc GVB
MW309584 15-07 Grillo GVB
MW309598 21-03 Mazuelo Nero GVB 2
MW309582 15-01 Graciano GVB 1
MW309558 05-02 Azal branco GVB
MW309560 05-03 Azal tinto GVB 2
MW309574 10-15 Cape Corinth GVB
MW309603 22-03 Muscat d Alexandrie blanc Pear berry GVB
OQ999891 03-15 Alicante bouchet GVB 2
MW309635 48-03 Auxerrois GVB
MW309636 48-09 Tinta Amarela GVB
KX522545 GVB 248 South Africa

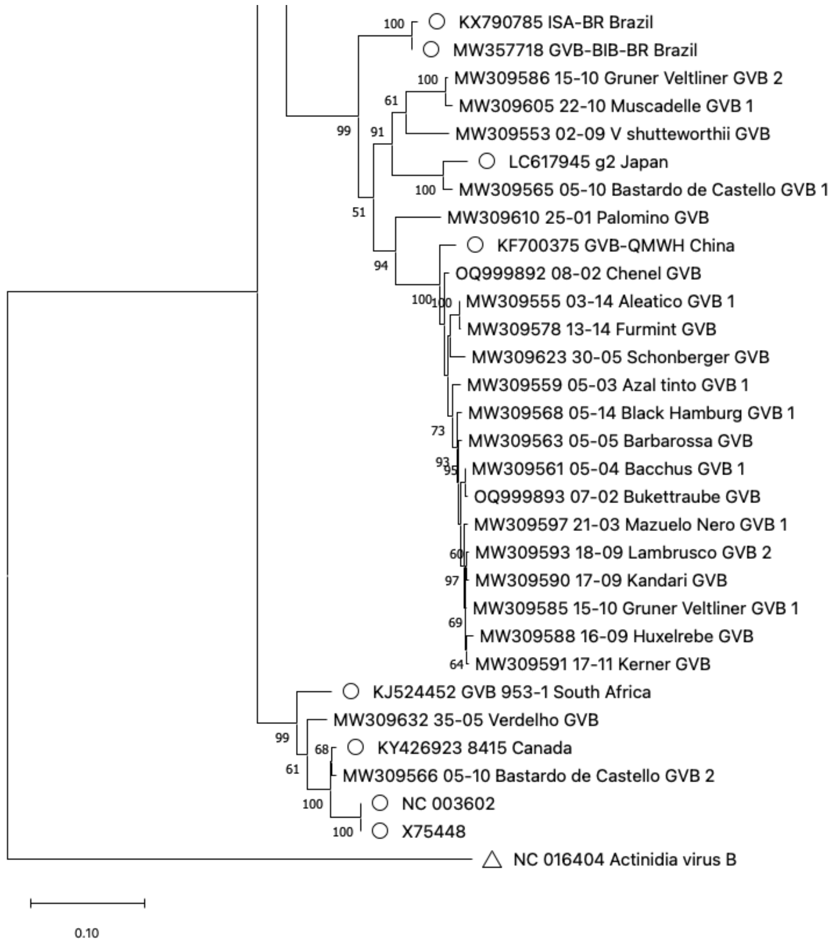

Figure S5: Maximum likelihood phylogeny based on the amino acid sequences of the RNA-dependant RNA polymerase (RdRp) gene of grapevine virus B (GVB) from this study together the cognate sequences from selected variants of GVB from GenBank (indicated by open circles). The cognate RdRp sequence from actinidia virus B was used as an outgroup (indicated by an open triangle). The phylogeny represents the tree with the highest log likelihood and was generated in MEGA X (Stecher et al. 2020) using the Jones-Taylor-Thornton (JTT) (Jones, W. R. Taylor, and J. M. Thornton. (1992) with gamma distribution (n=5) and invariant sites. Bootstrapping was applied (1000 replicates) and the percentage of trees in which the associated taxa clustered together is shown next to the branches. Bootstrap percentages lower than 50 are not shown.
