# Supplementary material for: Metaviromic Characterization of Betaflexivirus Populations Associated with a Vitis cultivar Collection in South Africa
Source: Viruses. 2023 Jun 29;15(7):1474. doi: 10.3390/v15071474 (PMC10385141; doi:10.3390/v15071474)
Supplement: Supplementary file 1 [file viruses-15-01474-s001.zip › Figure S8.pdf]

		1	2	3	4	5	6	7	8	9	10	11	12	13	14	15	16	17	18	19	20	21	22	23	24	25	26	27	28
02-01_V_flexuisa_GVE	1		5205	5131	5208	5210	5206	5213	5211	5212	5165	5206	5060	5207	5167	4876	5212	5160	5208	5209	5210	5174	5191	5205	5213	5205	5208	5196	5195
15-14_Hebron_GVE	2	68,50		7384	7443	7446	7448	7453	7448	7442	7395	7445	7287	7449	7413	7051	7449	7394	7450	7454	7453	7421	7389	7393	7399	7396	7386	7388	7393
16-15_Heroldrebe_GVE	3	67,52	97,59		7427	7437	7443	7443	7445	7437	7435	7440	7366	7444	7444	7130	7436	7435	7445	7442	7440	7445	7309	7321	7319	7322	7308	7314	7322
30-05_Schonberger_GVE	4	68,54	98,37	98,21		7513	7525	7530	7523	7525	7474	7522	7362	7524	7492	7129	7524	7473	7529	7528	7526	7500	7391	7404	7400	7403	7394	7391	7397
14-13_Gamay_Noir_GVE	5	68,55	98,35	98,23	99,23		7536	7536	7539	7529	7476	7529	7370	7535	7497	7134	7532	7471	7528	7534	7534	7503	7378	7387	7400	7395	7383	7383	7388
28-10_Rotberger_GVE	6	68,51	98,43	98,36	99,44	99,52		7551	7554	7538	7484	7541	7380	7547	7509	7146	7543	7483	7542	7545	7543	7515	7384	7391	7400	7399	7387	7387	7394
30-08_Semillon_GVE	7	68,60	98,45	98,32	99,47	99,54	99,74		7556	7541	7486	7540	7380	7546	7510	7146	7546	7487	7545	7547	7548	7514	7385	7398	7407	7402	7390	7389	7399
25-09_Poulsaid_GVE	8	68,57	98,38	98,34	99,37	99,58	99,76	99,80		7541	7484	7541	7382	7547	7509	7146	7544	7485	7542	7546	7548	7513	7383	7391	7404	7400	7385	7386	7394
08-09-Chardonnay_GVE	9	68,59	98,31	98,24	99,41	99,45	99,56	99,62	99,60		7476	7533	7372	7543	7501	7140	7535	7473	7534	7536	7537	7503	7377	7386	7398	7393	7383	7380	7388
05-10_Bastardo_de_Castello_GVE	10	67,97	97,74	98,97	98,84	98,75	98,90	98,89	98,85	98,76		7485	7372	7485	7485	7138	7483	7481	7488	7489	7487	7492	7335	7344	7345	7348	7336	7337	7345
07-15_Cataratto_GVE	11	68,51	98,40	98,36	99,44	99,45	99,66	99,60	99,60	99,51	98,96		7385	7548	7514	7152	7536	7482	7537	7542	7540	7512	7381	7394	7399	7400	7386	7387	7393
04-15_Aligote_GVE	12	66,59	96,31	98,46	97,36	97,35	97,53	97,49	97,50	97,38	98,14	97,63		7387	7393	7153	7375	7374	7380	7379	7379	7382	7224	7233	7237	7240	7225	7230	7239
11-05_Delight_GVE	13	68,52	98,45	98,39	99,44	99,52	99,74	99,68	99,68	99,64	98,93	99,76	97,63		7516	7155	7540	7484	7541	7546	7544	7514	7383	7394	7401	7400	7388	7391	7397
05-15_Black_Monukka_GVE	14	68,00	97,98	98,88	99,07	99,02	99,23	99,21	99,18	99,09	99,43	99,34	98,21	99,34		7156	7502	7486	7511	7508	7506	7511	7349	7360	7363	7366	7354	7355	7363
05-14_Black_Hamburg_GVE	15	64,17	93,19	95,31	94,27	94,23	94,44	94,40	94,39	94,32	95,02	94,55	96,62	94,57	95,06		7139	7138	7146	7145	7143	7148	6992	7001	7008	7011	6996	7002	7009
17-07_Kadarka_GVE	16	68,57	98,38	98,20	99,37	99,47	99,60	99,66	99,63	99,51	98,82	99,52	97,40	99,58	99,08	94,28		7502	7539	7545	7552	7516	7383	7392	7403	7398	7386	7386	7393
16-09_Huxelrebe_GVE	17	67,90	97,73	98,99	98,82	98,68	98,89	98,90	98,86	98,72	99,59	98,92	98,18	98,92	99,44	95,03	99,08		7489	7488	7492	7493	7330	7341	7344	7347	7333	7334	7342
18-11_Lagrein_GVE	18	68,54	98,47	98,45	99,56	99,43	99,67	99,67	99,62	99,52	99,02	99,64	97,59	99,67	99,33	94,50	99,56	99,03		7543	7541	7517	7388	7398	7398	7401	7391	7390	7401
23-15_Muscat_St_Laurent_GVE	19	68,55	98,51	98,35	99,48	99,51	99,70	99,70	99,67	99,55	98,97	99,67	97,52	99,72	99,22	94,42	99,64	98,96	99,68		7549	7518	7387	7397	7404	7402	7389	7392	7397
31-11_Seneca_GVE	20	68,56	98,45	98,28	99,42	99,51	99,63	99,71	99,70	99,56	98,90	99,60	97,48	99,66	99,15	94,36	99,74	98,97	99,62	99,72		7520	7383	7397	7405	7400	7388	7389	7395
35-14_Riesling_GVE	21	68,09	98,08	98,86	99,18	99,10	99,31	99,26	99,23	99,11	99,48	99,31	98,02	99,31	99,73	94,91	99,26	99,50	99,40	99,35	99,34		7359	7368	7369	7372	7362	7362	7369
10-04_Crouchen_blanc_GVE	22	68,31	97,66	96,65	97,74	97,45	97,58	97,56	97,52	97,45	97,00	97,58	95,53	97,58	97,18	92,46	97,50	96,93	97,70	97,62	97,53	97,32		7400	7410	7410	7379	7372	7386
03-13_Alden_GVE	23	68,50	97,71	96,81	97,91	97,57	97,67	97,73	97,62	97,57	97,12	97,75	95,65	97,73	97,33	92,58	97,62	97,08	97,83	97,75	97,71	97,43	97,86		7411	7410	7389	7378	7404
54-13_Merzling_GVE	24	68,60	97,74	96,68	97,75	97,74	97,74	97,85	97,79	97,73	97,03	97,74	95,60	97,77	97,27	92,58	97,77	97,01	97,73	97,81	97,82	97,34	97,89	97,90		7554	7390	7393	7396
44-02_Katta_Khurgan_GVE	25	68,50	97,75	96,83	97,90	97,68	97,78	97,78	97,74	97,66	97,17	97,83	95,74	97,81	97,41	92,71	97,70	97,16	97,87	97,82	97,75	97,49	97,99	97,99	99,79		7389	7392	7395
03-03_Affenthaler_GVE	26	68,54	97,62	96,64	97,78	97,52	97,62	97,62	97,54	97,53	97,01	97,65	95,54	97,65	97,25	92,52	97,54	96,97	97,74	97,65	97,60	97,36	97,58	97,71	97,62	97,71		7394	7391
10-07_Cornichon_violet_GVE	27	68,38	97,65	96,72	97,74	97,52	97,62	97,61	97,56	97,49	97,02	97,66	95,61	97,69	97,26	92,59	97,54	96,98	97,73	97,69	97,61	97,36	97,49	97,57	97,66	97,75	97,78		7413
11-03_David_GVE	28	68,36	97,71	96,83	97,82	97,58	97,71	97,74	97,66	97,60	97,13	97,74	95,73	97,77	97,37	92,69	97,64	97,09	97,87	97,75	97,69	97,45	97,67	97,91	97,70	97,79	97,74	98,03	

Figure S8: Average nucleotide identity comparisons between the complete/near complete genomes of grapevine virus E variants from this study. The x-axis values express average nucleotide variability between variants as a percentage, while the y-axis values present the total number of nucleotide differences between each variant.
