# Supplementary material for: Metaviromic Characterization of Betaflexivirus Populations Associated with a Vitis cultivar Collection in South Africa
Source: Viruses. 2023 Jun 29;15(7):1474. doi: 10.3390/v15071474 (PMC10385141; doi:10.3390/v15071474)
Supplement: Supplementary file 1 [file viruses-15-01474-s001.zip › Figure S9.pdf]

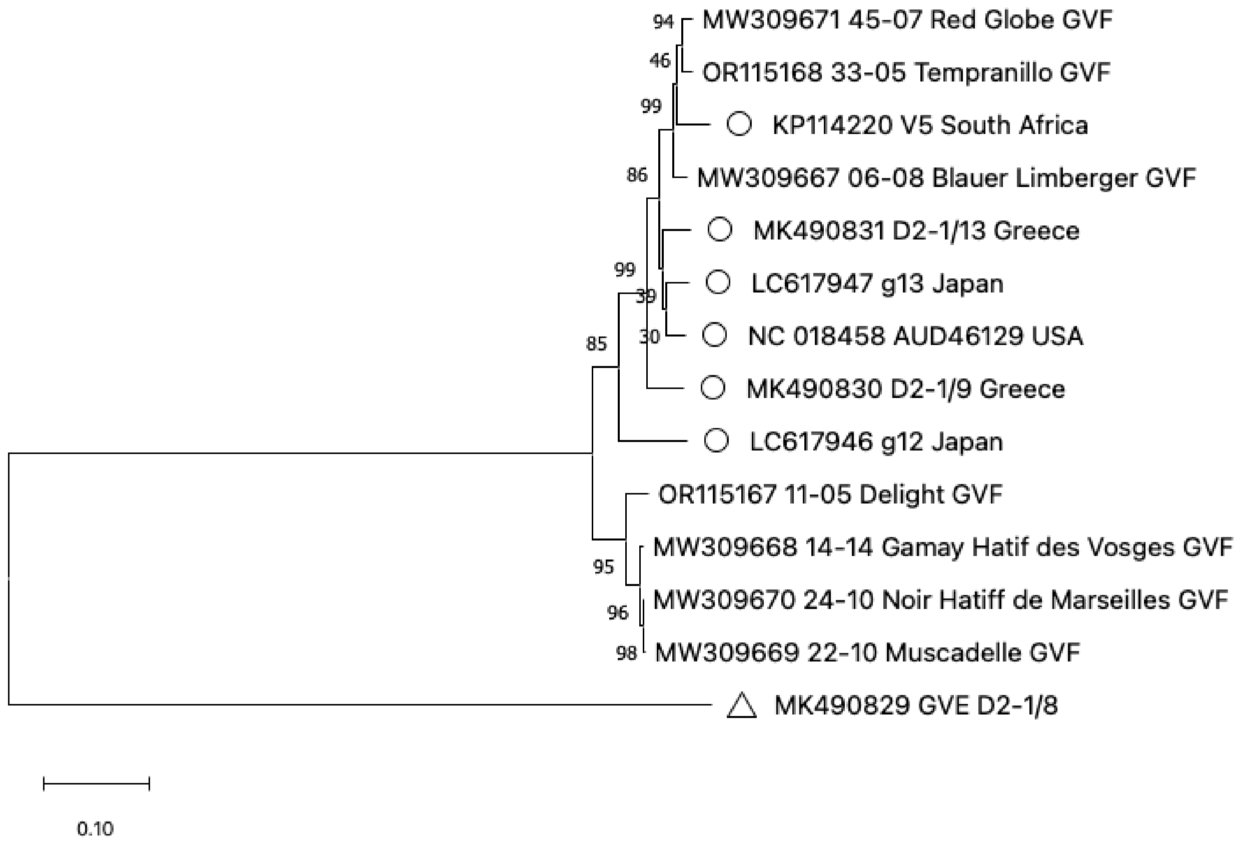

Figure S9: Maximum likelihood phylogeny based on the amino acid sequences of the RNA-dependant RNA polymerase (RdRp) gene of grapevine virus F (GVF) from this study together the cognate sequences from selected variants of GVF from GenBank (indicated by open circles). The cognate RdRp sequence from grapevine virus E was used as an outgroup (indicated by an open triangle). The phylogeny represents the tree with the highest log likelihood and was generated in MEGA X (Stecher et al. 2020) using the Le Gascuel model Le and Gascuel (2008) with gamma distribution (n=5). Bootstrapping was applied (1000 replicates) and the percentage of trees in which the associated taxa clustered together is shown next to the branches. Bootstrap percentages lower than 50 are not shown.
